# Germline variant screening with targeted next generation sequencing in prostate cancer: phenotype-genotype correlation

**DOI:** 10.3906/sag-2105-348

**Published:** 2021-09-27

**Authors:** Ali Yavuz ÇAKIR, Kuyaş HEKİMLER ÖZTÜRK, Alper ÖZORAK

**Affiliations:** 1 Department of Bioengineering, Science Institute, Süleyman Demirel University, Isparta, Turkey; 2 Department of Medical Genetics, Faculty of Medicine, Süleyman Demirel University, Isparta, Turkey; 3 Department of Urology, Faculty of Medicine, Süleyman Demirel University, Isparta, Turkey

**Keywords:** Next generation sequencing, bioinformatics, prostate cancer, databases, germline mutations

## Abstract

**Background/aim:**

Next generation sequencing provides new information about the molecular pathogenesis of cancer. We used a targeted NGS-based multiple gene panel comprising prostate cancer (PCa) predisposing genes to assess the prevalence of germline mutations in PCa patients.

**Material and methods:**

In a cohort of twenty-one PCa patients with a family history of cancer, a targeted multigene panel consisting of 39 genes associated with hereditary cancer was created and analyzed using the next generation sequencing method. The novel and pathogenic mutations detected were confirmed by Sanger sequencing method. Thereafter, the data obtained were evaluated using different genomic variant classifiers and databases.

**Results:**

With an incidence of less than 5% in different populations (MAF<0.05); a total of 81 variants were identified, including 41 missense, 16 synonymous, 3 splice-site, 11 intronic, 5 in-del and 5 novels. According to the ACMG criteria, 5 (6.2%) of these variants are pathogenic/likely pathogenic; 5 (6.2%) of them were classified as novel variants. In addition, variants having very low-frequency and unknown clinical significance (VUS) in the databases were detected.

**Conclusion:**

The findings we obtained from this study contributed to the understanding the genetic pathogenesis of PCa, determining the frequency of mutations in the population, and revealing the genotype-phenotype correlations. Additionally, we demonstrated that using multigene panel-based genetic tests rather than single-gene tests in germline mutation screening in hereditary PCa will be more beneficial in terms of genetic counseling.

## 1. Introduction

The genetic etiology of prostate cancer (PCa) is complex and poorly understood. Furthermore, there are multiple predisposing factors that can also affect severity, progression, and the outcome of PCa [1]. Genetic changes that are known as copy number variations, point mutations, small insertions or deletions, structural rearrangements, and chromosomal aberrations are generally involved in carcinogenesis, which can be in germline DNA or tumor genome [2].

Germline (i.e. inherited) mutations that predispose to hereditary cancers have become the focus of many studies; therefore, they have the potential to predict both incidence and prognosis. In addition, these variations have important implications in areas such as staging, screening, treatment, genetic counselling, and cascade testing of family members and, hence, can serve as therapeutic targets [3]. However, diverse ethnic population, geographic heterogeneity, the presence of rare variants, and incomplete family histories create limitations to use these variations for this purpose. Therefore, it is important to overcome these limitations as hereditary risk of PCa has been associated with a higher Gleason score, metastases at diagnosis, and poor prognosis [4, 5]. According to the NCCN guidelines (Prostate Cancer, Version 2.2019), positive family history increases the risk of developing this disease and according to studies approximately 11% of patients with PCa, and at least one additional primary cancer carries germline mutation associated with increased cancer risk. Consequently, the relevant guideline recommends that all patients with PCa should be carefully examined in terms of their own information and family histories [6].

With the advancement of next generation sequencing (NGS) technologies, simultaneous sequencing of cancer susceptibility genes has been achieved and has become a more effective genetic testing strategy compared to single gene testing [7]. These recent advances in NGS technology have, therefore, both enabled us to better understand the biology of prostate tumors as well as and supported an understanding of the genetic basis of the clinical variability of the disease and an orientation towards a personalized treatment paradigm [3, 7].

In this study, germline mutation screening application was performed with multi-panel tests. With multi-panel tests, germline mutation screening are well-established diagnostic tools to identify the origin of cancer clusters in a family. They also provide early diagnosis and implementation of the most appropriate preventive measures. Patients with PCa who also have a familial history of cancer were included in this study and DNA repair genes as well as genes associated with PCa in GWAS studies were sequenced with targeted next generation sequencing method, which aimed to correlate with the connection of the detected mutation, single nucleotide polymorphism (SNP), small deletions and insertions with the help of databases. In addition, it is also aimed to identify new gene variants, determine pathogenic, clinical significance of unknown (VUS), novel variants frequencies, and establish genotype-phenotype correlations.

## 2. Material and methods

### 2.1. Patient data

The study was initiated with the decision of Süleyman Demirel University Faculty of Medicine Clinical Research Ethics Committee (Date 11.03.2019, No:92). Written informed consent was obtained from all the patients. Twenty-one patients, between the ages of 45 and 75 (mean age 64.7 ± 7.9 years) who were diagnosed with PCa and had a family history of cancer applied to Süleyman Demirel University Faculty of Medicine, Urology policlinic, were included in the study. Volunteers meeting the study criteria in patients who applied to the urology policlinic were randomly selected and recruited. Patients with malignancies other than PCa were excluded from the study. Prostate-specific antigen (PSA) levels (mean 37.9 ± 41.4) were evaluated in the serum of the patients. Histopathological grading was done according to the Gleason score (GS) grading methods.

### 2.2. Extraction of genomic DNA

Genomic DNA (gDNA) was extracted from peripheral blood using a MagPurix Blood DNA Extraction Kit. DNA was isolated from a 200-μL blood sample using Zinexts MagPurix system (Zinexts Life Science Corp., New Taipei City, Taiwan) according to the manufacturer’s protocol.

### 2.2. Multi-gene panel testing using targeted NGS

DNA libraries were generated by the target exon-capture method. Paired-end sequencing was performed on the Illumina MiSeq NGS System (Illumina Inc, San Diego, CA, USA) using the MiSeq Reagent Nano Kit v2 (500 cycles) (Catalog No: MS-103-1003, Illumina Inc., San Diego, CA, USA). In this study, all the coding exons ±25 bp from each direction of 39 PCa associated genes (*AKT1, APC, AR, ATM, BARD1, BRCA1, BRCA2, BRIP1, CDKN2A, CHEK2, EHBP1, ELAC2, EPCAM, EPHB2, FANCA, FGFR4, GREM1, HNF1B, HOXB13, IGF2, ITGA6, MLH1, MSH2, MSH6, MSMB, MSR1, MUTYH, NBN, PALB2, PLXNB1, PMS2, POLD1, POLE, RAD51C, RAD51D, RNASEL, STK11, TP53, WT1*) were sequenced by using hybridization-based targeted genomic sequencing (Celemics, Inc., South Korea). On average, 29% of the annotations obtained are within the target area (annotation on Target-CDS±25 bp) and 71% of them are outside the target area (annotations off target). In the study, the ratio of the target region covered above the depth of 20X was 98.30%, and the ratio of the target region covered at a depth of 50X and above was 88.55%.

### 2.3. Sequencing data analysis, filtering criteria

Our bioinformatics analysis involves 5 steps: fastq quality control, sequence alignment (Burrows-Wheeler Alignment (BWA)-MEM Tool), post-alignment processing (MarkDuplicates), variant calling (Freebayes), and downstream analyses. As the first step of downstream analysis, we filtered variants according to the population allele frequency and discarded the variants that were seen more than 5% frequency in any given databases. In addition to the population databases, the synonymous variants were not included in the variant results. Various bioinformatics tools were applied for variant calling, depending on the current germline mutation calling analysis pipelines. The read quality of the high throughput sequence data obtained as FASTQ raw data was assessed using the FASTQC (Babraham Bioinformatics) program. The data obtained were aligned to the human reference genome GRCh37.p12 with the BWA-MEM tool, which works with the local alignment algorithm. Low-quality data (reads) were trimmed with the Trimmomatic tool and duplicated reads were filtered with the MarKDuplicates tool since hybridization-based kits were used in the study. As a result of the study, reads between 150K and 500K were obtained per sample. On average, for each sample, 10% of the reads could not be aligned. In our study: Clinvar dated 2021-04-18 and dbSNP dated 2020-04-21 (v154) were used. The Het/Hom ratio was used in the quality control phase. In the study of Guo et al., it is stated that Het/Hom ratio should be 2.0 in WGS studies based on Hardy-Weinberg equilibrium [8]. The Het/Hom ratio of our study is 2.2. The coverage requirements for reporting were ≥20 unique reads (20X) for each base. In our study, the average read depth per base was found to be 218. The detected variants were verified with the IGV (integrative genomics viewer) program.

### 2.4. Evaluation of the pathogenicity of the variants

As a continuation of the downstream analysis, the detected genomic variants were annotated with the help of several databases and platforms. GnomAD, ExAC, 1000 Genomes, ESP6500 databases were used for the population frequencies of the variants we detected. In order to examine the effects of the variants, ClinVar, Varsome, and Franklin were used to evaluate the pathogenicity of the variants according to American College of Medical Genetics and Genomics (ACMG) classification. The detected genomic variants were annotated with the help of several databases and platforms.

### 2.5. Confirmation analysis

All variants detected as novel, pathogenic (P) and likely pathogenic (LP) obtained after the NGS study were confirmed by the Sanger 3500 Series Genetic Analyzers sequencing method (Applied Biosystem, ThermoFisher, Scientific, USA). DNA samples of 50 ng from the patients carrying these variants were amplified by PCR method with the targeted primers. Amplification products were paired-end sequenced with BigDye Terminator v3.1, in accordance with the manufacturer’s instructions. The data obtained were analyzed with SeqScape v3.0 and Sequencing Analysis v6.0 (ThermoFisher, Scientific, USA) software using the GRCh37/hg19 reference genome. Primer sequences are available under request.

## 3. Results

### 3.1. Identification of candidate variants

In 21 PCa samples examined in our study, the incidence of the variants in different populations was below 5%; a total of 81 variants were identified, including 41 missense, 16 synonym, 3 splice-site, 11 intronic, 5 in-del and 5 novel mutations. Percentage distribution of all the variants we found by genes are; *APC* (7%), *AR* (5%), *ATM* (2%), *BARD1* (1%), *BRCA1* (1%), *BRCA2* (9%), *BRIP1* (4%), *CDKN2A* (1%), *CHEK2* (4%), *EHBP1* (5%), *ELAC2* (2%), *EPHB2* (2%), *FANCA* (7%), *FGFR4* (4%), *ITGA6* (4%), *MSH2* (1%), *MSH6* (5%), *MSMB* (1%), *MSR1* (1%), *MUYH* (2%), *NBN* (1%), *PLXNB1* (7%), *POLE* (5%), *POLD1* (4%), *PMS2* (2%), *RAD51C* (2%), *RNASEL* (1%), *STK11* (1%), *TP53* (2%), *WT1* (2%) (Figure). The most variant was found in the *BRCA2* (9%) gene, followed by *APC* (7%), *FANCA* (7%) and *PLXNB1* (7%). Heterozygous variants c.497C>G in *APC*, heterozygous variants c.3887C>A in *APC*, heterozygous variants c.722-10T>C in *CHEK2*, heterozygous variants c.1638A>C in *FANCA*, heterozygous variants c.182+11_182+15delAGACCinsGGACT in *ITGA6* have not been previously defined in population databases, and it was evaluated as a novel mutation according to the ACMG criteria. Additionally, VUS and variants with very low frequency were detected in the databases.

Variants were not found in the *AKT1, CDKN2A, EPCAM, GREM1, HNF1B, HOXB13, IGF2, MLH1, PALB2, RAD51D*, and *RNASEL* genes. All variants (except for the *AR* gene homozygous c.1174C>T variant observed in one patient) were found to be heterozygous. The Human Genome Variation Society (HGVS) names, gnomAD, ExAC, ESp6500, 1000 Genome frequencies, and other features of all the filtered variants, which are missense and novel, were summarized in Table 1.

### 3.2. Variant classification and genotype-phenotype correlations

Variants were evaluated according to the recommendations of ACMG Standards and Guidelines. Germline mutations were detected in 18 out of 21 samples and mutations that meet the criteria were not found in 3 samples at all. Of the missense variants we detected classification was as the following: According to the VarSome database, 27 (60%) were B/LB, 13 (28.9%) were VUS, 5 were (11.1%) P/LP; according to the Franklin database, 18 (40%) were B/LB, 26 were (57.8%) VUS, 1 was (2.2%) P/LP; according to the ClinVar database, 21 (46.7%) were B/LB, 7 (15.6%) were VUS, 2 (4.4%) were P/LP, and 15 (33.3%) were NA. Pathogenic/likely pathogenic (P/LP) variants correspond to 6.2% of all variants meeting the criteria and these variants were seen in 28.5% of patients. According to VarSome, Franklin, and ClinVar databases; *BRIP1* (c.139C>G), *AR* (c.1174C>T and c.237_239delGCA), *TP53* (c.654C>T), *MUTYH* (c.2T>C) variants were classified as P/LP. Other variants were classified as benign, likely benign, and VUS. First-degree relatives of patients with P/LP had a history of cancer. Patients with P/LP and novel variants (mean age 59.78 ± 5.3) were diagnosed at a younger age than patients without these variants (mean age 68.4 ± 7.7). Of the patients carrying germline positive variant, 44.4% were metastatic prostate cancer, 5.6% clinical stage T2a, 5.6% clinical stage T2b, 16.7% clinical stage T2c, and 27.8% clinical stage T3a. P/LP variant was detected in 3 (14.3%) of 7 men with PCa with Gleason score of 6, and in 3 (21.4%) of 14 men with PCa with Gleason score of 7 and above. Serum PSA mean of patients with novel mutation was 19.57 ± 19.47, while the mean of serum PSA of patients with P/LP variant was 14.33 ± 17.66. Patients with high PSA values and metastatic cancer had at least 3 related germline mutations. Details of the missense and novel variants and clinical characteristics of patients with these variants are given in Table 2.

## 4. Discussion

This study is the first research that determines the spectrum of genes related to the disease in patients with PCa who have a familial history of cancer. In our study, we performed target capture sequencing by using a custom designed multigene panel to estimate the frequency of pathogenic and novel germline variant carriers in patients with PCa. It was found that 28% of patients had deleterious cancer susceptibility gene mutations. Additionally, 5 novel variants were identified that had never been previously described in the literature or reference databases. The tumor stage was not different between patients with and without deleterious mutations. However, the age at diagnosis was lower in patients with a positive deleterious mutation. It could also be said that individuals carrying variants in DNA repair genes are at risk of PCa, since both the most frequently mutated genes and genes with pathogenic/novel variants are associated with DNA repair functions.

Studies have stated that the probability of PCa occurring as a result of variants inherited from families is 37.5%. Thus, identifying a pathogenic variant in a PCa patient can provide many benefits [9]. In the literature, mostly *BRCA* genes have been taken into consideration in germline mutation screening studies in PCa. Although it was not found a P/LP variant in *BRCA* genes in this study, the most frequently mutated gene was *BRCA2* (9%). Due to the link between different cancer syndromes and common clinical outcomes, a wide range of candidate genes including *BRCA* genes was created in this study. Therefore, expanded NGS gene panels would be greatly useful not only for the clinical management of patients but also for identifying high-risk asymptomatic individuals in subsequent generations and relatives [10].

In this study, sequence analysis of 39 genes associated with PCa obtained a diagnostic yield of 28%. As a result, most pathogenic mutations were detected in the *AR* gene. The homozygous *AR* c.1174C>T pathogenic variant was found in a patient at diagnosis age of 52, at clinical stage T3a. Pathogenic variant of *AR* c.237_239delGCA was detected in 2 patients. The clinical stage of both patients was T3a, and the age of diagnosis was 55 and 62. This variant was previously detected in the group with testicular cancer, but no significant difference was found compared to the control group [11]. It was known that the variants in the *AR* gene which plays a role in the development of prostate tissue were associated with an increased risk of PCa [12]. Furthermore, it had been shown that the *AR* gene regulates the transcriptional mechanism of DNA repair genes [13]. The study in which the *AR* gene was sequenced in PCa in the literature was limited. It was reported these germline variants were detected in PCa patients for the first time in Turkey. The results in this study supported the necessity of including the *AR* gene in clinical genetic testing of PCa.

One P/LP germline mutation was detected in each of the *BRIP1, TP53, MUTYH* genes according to ClinVar and Varsome. The patient with *BRIP1* c.139C>G pathogenic variant was 62 years of age at diagnosis, the clinical stage metastasis, and Gleason score was 8 (4+4). This variant had not been previously reported in PCa. Moyer et al. [14] found ATPase deficiency and helicase activity deficiency in the protein of this variant in a functional study performed in breast cancer. In the literature, missense mutations in the *TP53* gene had been associated with the development of cancer, and it was suggested that PCa sensitivity develops in families carrying these variants [4]. The diagnosis age of patient with pathogenic *TP53* c.604C>T variant was 65 and clinical stage metastasis and gelason score was 7 (4+3). This variant had been previously reported in various cancers and had been classified as P/VUS [15]. Although the connection of the *MUTYH* gene with the risk of PCa was not clear, it was added to the test panel in this study as the protein product of the gene plays a role in repairing mismatches that occur in DNA replication. The population frequency of the missense *MUTYH* c.2T>C variant had not been previously reported. Leongamornlert et al. [16] reported that the *MUTYH* gene c.940C>T variant detected in a PCa patient was inherited in families and was associated with disease severity. In accordance with the literature, the clinical stage of the patients who carried the c.2T>C variant was T3a, and the age of diagnosis was 58. Considering the clinical data of patients with these P/LP variants, it could be said that it caused more aggressive progress. Although functional losses in these genes are associated with cancer, larger studies are needed to confirm this.

In this study, 5 novel variants in the *APC, CHEK2, FANCA, and ITGA6* genes were identified. These variants were missense, intronic, and indel variants. These variants, which were not found in the ClinVar database, were classified as VUS in the VarSome and Franklin databases. Through silico tools such as PolyPhen-2 and SIFT, the pathogenicity of these variants was predicted as damaging. There was no information in the literature regarding the variants of the *APC* gene which were c.497C>G and c.3887C>A. Nicolosi et al. [4] reported the frequency of *APC* gene variants to be 4.5% among those carrying germline mutations in their study of 3607 PCa patients. They also argued that the connection of *APC* with PCa was not clear [4]. In this study, the fact that the *APC* gene (7%) was the most frequently mutated gene and the Gleason score of patients carrying these novel variants were 8 (4+4) may explain the sensitivity of PCa. Sufficient evidence could not be found for the pathogenicity classification of the intronic *CHEK2* c.722_10T>C variant, which was not previously reported in databases. The Gleason score of the patient carrying this variant was 7 (4+3), and the age of diagnosis was 64. Paulo et al. [15] reported that variants in the *CHEK2* gene would cause activation impairment due to loss of DNA damage response and phosphorylation deficiency. In the literature, loss of function in the *FANCA* protein involved in homologous recombination repair had been associated with PCa [17]. The missense *FANCA* c.1638A>C variant detected in this study had not been reported in databases before. Therefore, sufficient data could not be reached to evaluate its pathogenicity. However, it was striking that the age of diagnosis of the patient carrying this variant was 48 years. The study conducted by Mamidi et al. [18] showed the effect of various somatic and germline mutations on the aggressiveness of PCa and stated that some germline mutations in the *ITGA6* gene had an effect on aggressiveness. The indel variant *ITGA6* c.182+11_182+15delAGACCinsGGACT had not been previously reported in databases. The age of diagnosis of the patient carrying this variant was 58, and his Gleason score was 6 (3+3). However, it was difficult to demonstrate the effect of the *ITGA6* variant on the phenotype as the patient also carried the pathogenic variant *MUTYH* c.2T>C. Although novel variants were important because they cause amino acid changes, their classification became impossible due to the lack of entries in population databases and the inability to perform segregation analyzes. For a better understanding of variants with unknown clinical significance, it was necessary to obtain allele frequencies by studying more case groups and to conduct functional studies.

Variants classified as B/LB due to their high allele frequencies by ACMG were detected in the genes which were investigated in our study. B/LB variant was seen in almost all (17/18) patients who were detected a missense variant. It has been shown in the literature that benign variants do not cause disease. Likely benign variants are not expected to have an effect on the disease, as well. However, scientific evidence is currently insufficient to conclusively prove this. Additional evidence is needed to substantiate this claim. However, the possibility that these variants may contribute to the disease should not be ignored. Segregation studies and functional characterization analyzes are required to confirm the possible effect of these variants on the disease [19, 20, 21].

This study certainly had some limitations. The first of these was the low number of cases. The second was the inclusion of patients over 70 years of age changes the prevelance of germline mutations. The third was that the NGS method was not able detect large insertions/deletions, epigenetic modifications, and copy number changes from the molecular mechanisms of cancer. The fourth was that only the probands were tested in this study. It was necessary to carry out segregation analyzes and functional studies in order to reveal the disease risk of the defined variants. Additionally, the absence of mutations despite a positive family history in some patients included in the study suggested that there were other genes to be discovered.

Since the genes sequenced in this study have been studied together for the first time in PCa in Turkey, novel and valuable information had been obtained in order to understand the genetic pathogenesis of the disease, revealing the frequencies of variants and genotype-phenotype relationships. Early detection of pathogenic variants with germline cancer genetic testing in the clinical management of PCa patients may improve prognosis and quality of life in patients in terms of screening family members at risk and encouraging pre-metastasis surgery in the patient. Furthermore, increasing the usability of germline mutation tests with multigene panels will provide opportunities for targeted therapies that can improve PCa patients.

## Figures and Tables

**Figure:**
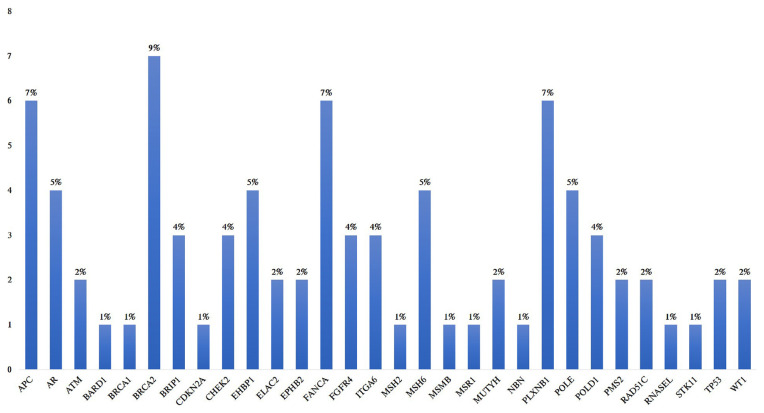
Percentage distribution of variants detected in PCa patients.

**Table 1:** Minor allel frequency and classification of variants identified in patients with PCa.

Gene	HGVS	Protein change	Genomic location GRCh37(hg19)	Zygosity	GnomAD Exome	ExAC Total	ESP6500 Total	1000 Genomes	dbSNP ID
***APC***	ENST00000457016.1: c.445G>C	p.Asp149His	chr5:112775651	Het	-	-	-	-	rs767875993
	ENST00000457016.1: c.497C>G	p.Thr166Ser	chr5:112111400	Het.	-	-	-	-	Novel mutation
	ENST00000457016.1: c.3887C>A	p.Ala1296Glu	chr5:112175178	Het.	-	-	-	-	Novel mutation
	ENST00000457016.1: c.7504G>A	p.Gly2502Ser	chr5:112178795	Het.	0.02	0.02	0.014	0.0008	rs2229995
***AR***	ENST00000374690.3: c.237_239delGCA	p.Gln80del	chrX:66765171	Het.	0.18	0.1	-	-	rs3032358
	ENST00000374690.3: c.1174C>T	p.Pro392Ser	chrX:66766162	Hom.	0.0041	0.0051	-	0.0074	rs201934623
	ENST00000374690.3: c.1406_1420del	p.Gly469_Gly473del	chrX:66766381	Het.	0.01	0.0012	-	-	rs746853821
***ATM***	ENST00000278616: c.5558A>T	p.Asp1853Val	chr11:108304736	Het.	0.000909	0.000008	-	0.0018	rs1801673
***BARD1***	ENST00000260947: c.1670G>C	p.Cys557Ser	chr2:214752454	Het.	0.02384	-	0.002724	0.008	rs28997576
***BRCA1***	ENST00000471181: c.4946T>C	p.Met1649Thr	chr17:43071031	Het.	0.000349	-	0.000227	0.0026	rs4986854
***BRCA2***	ENST00000380152: c.5312G>A	p.Gly1771Asp	chr13:32339667	Het.	0.000582	-	-	0.0002	rs80358755
	ENST00000380152: c.5744C>T	p.Thr1915Met	chr13:32340099	Het.	0.02907	-	0.005674	0.0086	rs4987117
	ENST00000544455.1: c.5785A>G	p.Ile1929Va	chr13:32914277	Het.	0.0008	0.00095	0.00007	0.013	rs79538375
	ENST00000544455.1: c.4258G>T	p.Asp1420Tyr	chr13: 32912750	Het.	0.0066	0.0067	0.0039	0.0039	rs28897727
	ENST00000544455.1: c.2918C>G	p.Ser973Trp	chr13: 32911410	Het.	0.000004021	**-**	**-**	**-**	rs397507296
***BRIP1***	ENST00000259008.2: c.139C>G	p.Pro47Ala	chr17:59937223	Het.	0.0002	0.0002	0.0002	-	rs28903098
***CDKN2A***	ENST00000498124.1: c.442G>A	p.Ala148Thr	chr9:21970916	Het.	0.0208	0.0223	0.0225	0.0069	rs3731249
***CHEK2***	ENST00000382580.2: c.623G>A	p.Gly208Asp	chr22:29121063	Het.	-	-	-	-	rs876660846
	ENST00000382580.2: c.722-10T>C	-	chr22:29115483	Het.	--	--	--	--	Novel mutation
***EHBP1***	ENST00000263991.5: c.2303A>G	p.Tyr768Cys	chr2:63176179	Het.	0.0021	0.0021	0.0018	0.0021	rs140508263
	ENST00000263991.5: c.2674G>C	p.Ala892Pro	chr2:63206431	Het.	0.000028	0.000033	-	-	rs754583745
	ENST00000263991.5: c.1976G>A	p.Ser659Asn	chr2:63175852	Het.	0.00047	0.00046	0.00038	-	rs140493234
***ELAC2***	ENST00000338034.4: c.1621G>A	p.Ala541Thr	chr17:12899902	Het	0.035	0.032	0.028	0.023	rs5030739
***EPHB2***	ENST00000400191.7: c.1081A>G	p.Ile361Val	chr1:22864990	Het	0.000581	-	-	0.0004	rs56180036
***FANCA***	ENST00000389301.3: c.3653C>T	p.Pro1218Leu	chr16:89809320	Het.	-	-	-	-	rs771111655
	ENST00000389301.3: c.3031C>T	p.Arg1011Cys	chr16:89818581	Het	0.00019	0.00013	0.000077	-	rs142377616
	ENST00000389301.3: c.2574C>G	p.Ser858Arg	chr16:89833576	Het	0.01	0.01	0.005	0.0093	rs17233141
	ENST00000389301: c.1638A>C	p.Gln546His	chr16:89846354	Het.	-	-	-	-	Novel mutation
***FGFR4***	ENST00000292408.4: c.1276G>A	p.Gly426Ser	chr5:176520431	Het.	0.000096	0.000058	0.000077	0.0002	rs55879131
***ITGA6***	ENST00000409080.1: c.182+11_182+15del AGACCinsGGACT	-	chr2: 173292709	Het.	-	-	-	-	Novel mutation
***MSH6***	ENST00000234420.5: c.2633T>C	p.Val878Ala	chr2:48027755	Het.	0.0106	0.0052	0.0055	0.0039	rs2020912
	ENST00000234420.5: c.663A>C	p.Glu221Asp	chr2:48025785	Het.	0.0007	0.0006	0.0008	-	rs41557217
	ENST00000234420.5: c.3151G>A	p.Val1051Ile	chr2:48028273	Het.	0.0009	0.0009	0.001	-	rs576269342
***MUTYH***	ENST00000450313.1: c.1544C>T	p.Ser515Phe	chr1:45795084	Het.	0.0085	0.0078	0.011	0.0045	rs140118273
	ENST00000450313.1: c.2T>C	p.Met1Thr	chr1:45805925	Het.	-	-	-	-	rs865954220
***PLXNB1***	ENST00000358536.4: c.4880G>A	p.Arg1627His	chr3:48454004	Het.	0.000036	0.000008	-	-	rs746314397
	ENST00000358536.4: c.866A>G	p.His289Arg	chr3:48465155	Het.	0.000004	-	-	-	rs1314691032
***POLE***	ENST00000320574.5: c.6494G>A	p.Arg2165His	chr12:133202740	Het	0.0058	0.0059	0.0067	0.0097	rs5745068
	ENST00000320574.5: c.2963C>T	p.Ser988Leu	chr12:133237652	Het.	0.00004	0.0001	-	0.0002	rs138391248
	ENST00000320574.5: c.2083T>A	p.Phe695Ile	chr12:133245032	Het	0.011	0.011	0.01125	0.0077	rs5744799
***PMS2***	ENST00000265849.7: c.1711C>A	p.Leu571Ile	chr7:6026685	Het.	0.007	0.0025	0.0057	0.0063	rs63750055
***RAD51C***	ENST00000337432.4: c.376G>A	p.Ala126Thr	chr17:56772522	Het.	0.00351	0.00347	0.00461	0.00199	rs61758784
***RNASEL***	ENST00000367559.3: c.196G>A	p.Ala66Thr	chr1:182555746	Het.	0.00011	0.00012	-	-	rs745739936
***TP53***	ENST00000269305: c.604C>T	p.Arg202Cys	chr17:7578245	Het.	0.000028	0.000016	-	-	rs587780072
***WT1***	ENST00000332351.3: c.1109G>T	p.Arg370Leu	chr11:32417943	Het.	0.00002	0.000008	-	-	rs554416372

NA, Not Available; HGVS, Human Genome Variation Society; Het, Heterozygous; Hom, Homozygous; ExAC, Exome Aggregation Consortium; ESP6500, Exome Sequencing Project; 1000Genomes, 1000 Genomes Project; GnomAD, The Genome Aggregation Database.

**Table 2:** Prioritized variants identified in the PCa cohort and its pathogenicity prediction.

Sample ID	Serum PSA	Gleason Score	TNM	Gene	dbSNP	Functional consequeces	VarSome	Franklin	ClinVar, UniProt	Variant Status	Clinvar Phenotype
1	10	3+4	T2b	CDKN2A c.442G>A	rs3731249	Missense	B	B	B	Known	-
				CHEK2 c.722-10T>C	NA	Intronic	VUS	VUS	NA	Novel mutation	-
				MSH6 c.2633T>C	rs2020912	Missense	B	B	B	Known	Colorectal cancer / Endometrial cancer, Lynch syndrome
				RNASEL c.196G>A	rs745739936	Missense	VUS	VUS	NA	Known	-
2	8.72	3+3	T2c	BRCA1 c.4946T>C	rs4986854	Missense	B	B	B	Known	Hereditary cancer, Breast-ovarian cancer
				BRCA2 c.5785A>G	rs79538375	Missense	LB	B	B	Known	Breast-ovarian cancer, Hereditary cancer
				FANCA c.3653C>T	rs771111655	Missense	LB	VUS	NA	Known	-
3	100	4+5	M	AR c.1406_1420del	rs746853821	Delesyon	VUS	B	LB	Known	-
				FGFR4 c.1276G>A	rs55879131	Missense	LB	VUS	NA	Known	-
6	80	4+5	M	BRCA2 c.4258G>T	rs28897727	Missense	B	B	B	Known	Breast cancer, Hereditary cancer
				EHBP1 c.2303A>G	rs140508263	Missense	VUS	VUS	NA	Known	-
				POLE c.6494G>A	rs5745068	Missense	LB	B	B	Known	Colorectal cancer, Hereditary cancer
7	50	4+4	M	APC c.497C>G	NA	Missense	LB	VUS	NA	Novel mutation	-
				BRCA2 c.2918C>G	rs397507296	Missense	VUS	VUS	NA	Known	Hereditary cancer
				BRIP1 c.139C>G	rs28903098	Missense	P	VUS	VUS(14), LB(2), P(3)	Known	Breast cancer, Hereditary cancer
				EHBP1 c.2674G>C	rs754583745	Missense	VUS	VUS	NA	Known	-
				ELAC2 c.1621G>A	rs5030739	Missense	B	B	B	Known	Prostate Cancer
8	13	3+3	M	APC c.7504G>A	rs2229995	Missense	B	B	B	Known	-
				BARD1 c.1670G>C	rs28997576	Missense	B	VUS	B	Known	Breast cancer, Hereditary cancer
				MUTYH c.1544C>T	rs140118273	Missense	B	B	B	Known	MYH-Associated polyposis, Hereditary cancer
				PLXNB1 c.866A>G	rs1314691032	Missense	VUS	VUS	NA	Known	-
9	18	3+4	T3a	BARD1 c.1670G>C	rs28997576	Missense	B	VUS	B	Known	Breast cancer, Hereditary cancer
				BRCA2 c.4258G>T	rs28897727	Missense	B	B	B	Known	Breast cancer, Hereditary cancer
10	124	5+5	M	BRCA2 c.5744C>T	rs4987117	Missense	B	B	B	Known	
11	100	5+5	M	APC c.7504G>A	rs2229995	Missense	B	B	B	Known	-
				ATM c.5558A>T	rs1801673	Missense	B	VUS	LB(7), B(5), VUS(1)	Known	Hereditary cancer, Ataxia-telangiectasia syndrome
				MSH6 c.3151G>A	rs576269342	Missense	LB	B	VUS(4), LB(2), B(4)	Known	Hereditary cancer
12	11.89	3+3	T3a	APC c.7504G>A	rs2229995	Missense	B	B	B	Known	-
				AR c.1174C>T	rs201934623	Missense	P	VUS	P(2), VUS(1), B(1)	Known	Partial androgen insensitivity syndrome, Hypospadias
				EHBP1 c.1976G>A	rs140493234	Missense	LB	VUS	NA	Known	-
				POLE c.2963C>T	rs138391248	Missense	VUS	VUS	VUS	Known	Colorectal cancer
				WT1 c.1109G>T	rs554416372	Missense	B	VUS	VUS(1)	Known	Drash syndrome, Wilms tumor
				BRCA2 c.5312G>A	rs80358755	Missense	B	B	B	Known	Breast and/or ovarian cancer, Hereditary cancer
13	8	4+3	M	MSH6 c.663A>C	rs41557217	Missense	LB	LB	VUS(6), LB(7), B(6)	Known	Hereditary cancer, Lynch syndrome
				TP53 c.604C>T	rs587780072	Missense	LP	LP	VUS	Known	Li-Fraumeni syndrome, Hereditary cancer
14	7.65	4+3	T2a	FANCA c.3031C>T	rs142377616	Missense	LB	VUS	LB	Known	-
				FANCA c.2574C>G	rs17233141	Missense	B	VUS	LB	Known	Fanconi Anemia
				POLE c.6494G>A	rs5744799	Missense	LB	B	B	Known	Colorectal cancer, Hereditary cancer
16	4.48	3+3	T2c	APC c.445G>C	rs767875993	Missense	VUS	VUS	VUS	Known	Familial adenomatous polyposis, Hereditary cancer
				FANCA c.1638A>C	NA	Missense	VUS	VUS	NA	Novel mutation	-
				PMS2 c.1711C>A	rs63750055	Missense	B	B	B	Known	Hereditary cancer, Lynch syndrome
17	6	3+3	T3a	AR c.237_239delGCA	rs3032358	Deletion	LP	VUS	P(1), B(1)	Known	Bulbo-spinal atrophy X-linked
				PLXNB1 c.4880G>A	rs746314397	Missense	VUS	VUS	NA	Known	-
18	100	4+4	M	EPHB2 c.1081A>G	rs56180036	Missense	LB	LB	NA	Known	-
				POLE c.6494G>A	rs5744799	Missense	LB	B	B	Known	Colorectal cancer, Hereditary cancer
19	5.41	3+3	T3a	BRCA1 c.4946T>C	rs4986854	Missense	B	B	B	Known	Hereditary cancer, Breast-ovarian cancer
				ITGA6 c.182+11_182+15delAGACCinsGGACT	NA	Indel	VUS	VUS	NA	Novel mutation	-
				MUTYH c.2T>C	rs865954220	Missense	P	VUS	VUS	Known	Polyposis, Hereditary cancer
				RAD51C c.376G>A	rs61758784	Missense	B	B	B	Known	Fanconi anemia, Breast-Ovarian cancer
21	28	4+4	T2c	APC c.3887C>A	NA	Missense	LB	VUS	NA	Novel mutation	-
				BRCA2 c.5312G>A	rs80358755	Missense	B	B	B	Known	Breast and/or ovarian cancer, Hereditary ca.
				CHEK2 c.623G>A	rs876660846	Missense	VUS	VUS	VUS	Known	Breast cancer, Hereditary cancer
22	4.7	3+3	T3a	AR c.237_239delGCA	rs3032358	Deletion	LP	VUS	P(1), B(1)	Known	Bulbo-spinal atrophy X-linked
